# Surgical Outcomes of LigaSure Hemorrhoidectomy in the Elderly Population: A retrospective cohort study

**DOI:** 10.1186/s12876-021-01969-1

**Published:** 2021-10-29

**Authors:** Chuang-Wei Chen, Tzung-Ju Lu, Koung-Hung Hsiao

**Affiliations:** 1 Division of Colon and Rectal Surgery, Department of Surgery, Taipei Tzu Chi Hospital, Buddhist Tzu Chi Medical Foundation, No. 289, Jianguo Rd., Sindian City, Taipei County, 231 Taiwan (R.O.C.); 2 School of Medicine, Tzu Chi University, Hualien, Taiwan (R.O.C.)

**Keywords:** Hemorrhoids, LigaSure hemorrhoidectomy, Elderly, Complication

## Abstract

**Background:**

This study aims to assess the association between age and outcomes in patients undergoing hemorrhoidectomy.

**Methods:**

This is a population-based cohort study. A retrospectively collected database with consecutive patients whose symptomatic prolapsed hemorrhoids managed by the LigaSure hemorrhoidectomy between Jan. 2015 and May 2017 was reviewed. Among 1238 patients, 1075 were under 65 years old (group 1), and 163 were 65 years old or older (group 2). Both groups were compared regarding baseline characteristics and surgical outcomes.

**Results:**

All patients tolerated the whole course of the operation in the prone jackknife position without anesthetic-associated complications. There was no significant difference between these two groups regarding sex, hemorrhoids grade, operation time, duration of hospital stays, postoperative pain score, analgesic consumption, total postoperative complications, re-admission rate, reoperation rate and follow-up times. The multivariate logistic regression analysis that may contribute to postoperative complications revealed no significant difference for all complications between both groups.

**Conclusion:**

The LigaSure hemorrhoidectomy for elderly patients is safe and effective without significant difference in short-term operative outcomes and all complication rates, compared with younger patients.

## Background

Hemorrhoidal disease is one of the most common and annoying disorders worldwide. The prevalence of symptomatic hemorrhoidal disease had been reported around 4% of the population [1–3]. The majority of hemorrhoids can be managed with conservative treatment; however, hemorrhoidectomy is still the definitive treatment for those Grade III or Grade IV hemorrhoids [4–7]. Although the incidence of hemorrhoids has been reported to be the highest for 45–65 years older adults [1], many people in the elderly population also have hemorrhoidal symptoms. As we all know, the aging of the population is a global trend. The population aged over 65 accounts for about 15% of the population in Taiwan. In our clinical practice, many elderly adults are troubled by symptoms related to hemorrhoids; however, they may worry about whether the risk is too high if they are considering surgery. In Taiwan, the people have generally considered the elderly population when they are older than 65 years old. Also, the majority of people will be retired when they are 65 years old [8]. Thus, we did not analyze the very old age population in this study but took 65 years as the cutoff for the two groups. To the best of our knowledge, there are still no articles describing hemorrhoidectomy in the elderly population. Thus, the purpose this study aims to assess the safety and effectiveness of hemorrhoidectomy performed in younger and elderly populations.

## Materials and methods

### Ethics statement

This study was approved by the institutional review board (IRB) of Taipei Tzu Chi Hospital and registered under the Declaration of Helsinki (http://www.researchregistry.com). Furthermore, all the study protocols have been reported in line with the STROCSS criteria.

### Study population

Patients whose symptomatic prolapsed hemorrhoids were managed by the LigaSure hemorrhoidectomy in our hospital between January 2015 and May 2017 was retrospectively reviewed. Among these patients, 1075 were aged under 65 years (group 1), and 163 were aged 65 years old or older (group 2). Both groups were compared for baseline characteristics (sex, hemorrhoids grade, ASA grade, and preoperative hemoglobin) and surgical outcomes (operative time, postoperative pain score, analgesic consumption, hospital stay, follow-up times, complications, re-admission, and re-operation rates). Patients who had acute thrombosed or strangulated hemorrhoids, previous perianal surgery, recurrent cases, or any other anorectal disorders such as fistula or fissure were excluded.

The grading system (I-V) of the American Society of Anesthesiologists (ASA) was used to evaluate the general condition of the patients before surgery. If the patients had a history of cardiovascular disease and were receiving anticoagulants or antiplatelet drugs, they were instructed to discontinue taking the medication for at least 1 week before surgery. They did not restart the medication until 1 week after the operation.

### Anesthetic method and surgical technique

All patients were admitted in the morning and received surgery in the afternoon. Patients were given a sodium phosphate enema preoperatively, and all patients underwent surgery in the prone jackknife position under general intravenous anesthesia with perianal local anesthetic infiltration. The intravenous anesthetic drugs consisted of fentanyl 2 ml (50 ug/ml), midazolam 2–5 ml (1 mg/ml), and propofol 2–10 ml (10 mg/ml). After the patient was in deep sedation, the perianal infiltration with 40 ml of an anesthetic agent (20 ml 0.5% bupivacaine, 20 ml 1% xylocaine, and 0.2 ml epinephrine 1:1000 solution) was performed. The intravenous fluid was only used for adding anesthetic drugs and was discontinued soon after the operation. Thus, the volume of intravenous fluid is minimal for every patient in both groups. The exposure was achieved utilizing a large-sized Hill-Ferguson retractor. All operations were performed by colorectal surgeons with the same surgical method. The LigaSure hemorrhoidectomy was performed by initially using the cutting mode of the electrocautery to make a narrow V-shaped incision from the external component of the hemorrhoidal cushion to the mucocutaneous junction. A long-smooth forceps was used to grasp and lift up the hemorrhoidal plexus. The LigaSure impact device was then applied beneath the forceps for coagulation from the mucocutaneous junction to above the apex of the hemorrhoidal cushion. The mucocutaneous bridges were turned over to excise residual hemorrhoidal tissues. After removing the piles, a Ferguson closed hemorrhoidectomy was performed, followed by putting a ligature on the wound's pedicle and closing the wound using 4/0 vicryl sutures. Usually, it was necessary to perform excisions of three hemorrhoidal cushions. Thus, patients in both groups had three wounds located at left lateral, right anterior and right posterior anal positions. The anal packing was not performed after the operation.

### Postoperative management

The definition of the five postoperative complications in this study included: (1) Anal stenosis: patients have postoperative anal stenosis and cannot undergo anal digital examination and have symptoms such as difficulty in defecation and pain. (2) Delayed bleeding: unexpected bleeding occurs 24 h after the operation, and patients required emergency room or outpatient treatment. (3) Fecal impaction: the difficulty of postoperative defecation and patients need to excavate the feces through an enema or digital anus examination. (4) Urine retention: The difficulty of postoperative urination and require catheterization. (5) Wound infection: the wound has persistent redness, swelling, heat, and pain after the operation.

Postoperatively, all patients were prescribed oral metronidazole (250 mg four times daily) for 5 days and a bulk laxative (Normal 1 pk/day) for 2 weeks. Postoperatively, all patients were prescribed oral metronidazole (250 mg four times daily) for 5 days and a bulk laxative (Normal 1 pk/day) for 2 weeks. After the hemorrhoidectomy, oral metronidazole was used as it was previous revealed as a cheap, safe, and effective intervention for reducing postoperative pain referring [9, 10]. Oral diclofenac, 75 mg twice a day, was given for pain control. If patients could not tolerate the pain, morphine (5 mg IV per time) or ketoprofen (30 mg IM per time) was given as needed every 6 h. Patients were instructed to irrigate the anal wound with warm water or sitz baths three times a day, and after every bowel movement. The neomycin ointment was prescribed for topical uses. In the morning of each postoperative day during hospitalization, the patients were instructed to complete a subjective pain survey using a visual analogue scale (VAS) ranging from 0 (no pain) to 10 (the worst pain). Patients were not discharged from the hospital until their pain was considered tolerably with oral analgesics, and there were no postoperative complications. After discharge, patients returned to the outpatient clinic at 2-week intervals for at least 3 visits. All the patients received follow-up at OPD for at least 6 weeks. The digital examination was performed to detect any possible stenosis. Patients were instructed to return to the clinic if any problems occurred in the future.

### The primary and secondary outcomes

The primary outcome is focused on whether age is a risk factor correlated with complications after hemorrhoidectomy. Surgical outcomes, total complication rate, delayed bleeding, urine retention, anal stenosis, recurrence, wound infection, re-admission rate, and re-operation rate between two groups were compared. The secondary outcome is to examine possible risk factors of complications after hemorrhoidectomy.

### Statistical analysis

Due to the skew and non-normal distributions, the continuous variables were expressed by median with inter-quartile range (IQR). The comparison between groups for the continuous or ordinal variables (preoperative hemoglobin level, operation time, postoperative pain score, narcotic consumption, hospital stay, and follow-up times) were performed using the non-parametric Mann–Whitney test. For the other categorical variables, data were presented by count with percentage and the associations with age group and complications were performed using the Fisher’s exact test. The univariable and multivariable logistic regression models were used to evaluate the factors that may contribute to postoperative complications. The factors with *p* value < 0.1 in univariable logistic regression models were included in the multivariable logistic regression model based on the backward conditional method. The statistical analyses were performed by the Statistical Product and Service Solutions (SPSS) Statistics 25.0 (IBM Corporation, Armonk, New York). All tests are two-sided, and p-values less than 0.05 were considered as statistically significant.

## Result

### Comparison between the younger and elder patients

For baseline characteristics, the mean age was 46.43 and 70.36 years in group 1 and 2, respectively, and their difference was significant (*p* < 0.01). However, no statistically significant difference was detected in sex and hemorrhoids grade (*p* > 0.05) between these two groups. The elder patient group (group 2) had significantly higher ASA grade (ASA II and III: 86.5% vs. 61.9%, *p* < 0.001) and lower pre-operative hemoglobin level (medians of 13.4 vs. 13.7 g/dL, *p* = 0.03) than the younger patient group (group 1). For most of the surgical outcomes, there was no significant difference between these two groups, including operation time, postoperative pain score, frequency of ketoprofen or morphine injection postoperatively (FKMIP), hospital stay, follow-up times, re-admission rate, and re-operation rate. The total complication rates between group 1 and 2 were also similar (8.1% and 9.2%, *p* = 0.646). But the kinds of complications between age groups were different (*p* = 0.033). The most frequent complication in group 1 was delayed bleeding (65/1075, 6.0%) followed by urine retention (15/1075, 1.4%). The most frequent complication in group 2 was urine retention (7/163, 4.3%) followed by delayed bleeding (6/163, 3.7%). All patients tolerated the procedure well and there were no instances of major anesthetic associated complications such as respiratory or cardiovascular compromise in either group (Table 1).

**Table 1:** Comparisons between two age groups for baseline characteristics and surgical outcomes

		Age group		*p* value
		Group 1 (< 65) (n = 1075)	Group 2 (≥ 65) (n = 163)	
Baseline characteristics Median age (range)		46.43(16, 64)	70.36(65, 86)	< 0.01*
Sex	Female	537 (50.0%)	83 (50.9%)	0.867^a^
	Male	538 (50.0%)	80 (49.1%)	
Hemorrhoids grade	III	743 (69.1%)	112 (68.7%)	0.928^a^
	IV	332 (30.9%)	51 (31.3%)	
ASA grade	I	409 (38.0%)	22 (13.5%)	< 0.001*^a^
	II	655 (60.9%)	135 (82.8%)	
	III	11 (1.0%)	6 (3.7%)	
Preoperative hemoglobin (g/dl)		13.7 (12.5, 14.8)	13.4 (12.2, 14.5)	0.030* ^b^
Surgical outcomes				
Operation time (min)		18.0 (13.0, 25.0)	18.0 (13.0, 25.0)	0.498 ^b^
Postoperative pain score		3.0 (2.0, 5.0)	3.0 (2.0, 5.0)	0.139 ^b^
FKMIP	0	664 (61.7%)	109 (66.9%)	0.139 ^b^
	1	298 (27.7%)	43 (26.4%)	
	2	78 (7.3%)	10 (6.1%)	
	3	27 (2.5%)	1 (0.6%)	
	4	6 (0.6%)	0	
	5	2 (0.2%)	0	
Hospital stay (day)		2.0 (2.0, 2.0)	2.0 (2.0, 2.0)	0.608^b^
Follow-up times	3	897 (83.4%)	135 (82.8%)	0.835^b^
	4	147 (13.7%)	23 (14.1%)	
	5	29 (2.7%)	4 (2.5%)	
	6	2 (0.2%)	0	
	8	0	1 (0.6%)	
Complications		87 (8.1%)	15 (9.2%)	0.646 ^a^
Anal stenosis		2 (0.2%)	1 (0.6%)	0.033* ^a^
Delayed bleeding		65 (6.0%)	6 (3.7%)	
Fecal impaction		3 (0.3%)	1 (0.6%)	
Urine retention		15 (1.4%)	7 (4.3%)	
Wound infection		2 (0.2%)	0 (0.0%)	
Re-admission		30 (2.8%)	4 (2.5%)	> 0.999 ^a^
Re-operation		5 (0.5%)	1 (0.6%)	0.572 ^a^

Categorical variables were presented by count and percentage. Continuous variables were presented by median and *interquartile range* (IQR). ^a^Fisher’s exact test. ^b^Mann–Whitney *U* test. *P is significant. Postoperative pain score: the pain score on the morning of the 1st postoperative day, ketoprofen (30 mg IM per time), morphine (5 mg IV per time)

American Society of Anesthesiologists (ASA); Frequency of ketoprofen or morphine injection postoperatively (FKMIP); Intramuscular (IM); Intravenous (IV)

In group 2, one patient developed anal stenosis requiring reoperation with anoplasty. In group 1, three patients developed delayed bleeding requiring reoperation and two patients developed perianal fistula-abscess requiring reoperation with fistulotomy.

### Factors associated with complications

The associations of baseline and peri-operative characteristics with complications were shown in Table 2, where sex, hemorrhoids grade, postoperative pain score, operation time, and frequency of ketoprofen or morphine injection postoperatively were significantly associated with the occurrence of complications. Compared with the patients without complications, those patients with complications were males (*p* = 0.039) and had higher hemorrhoids grade (*p* < 0.001), higher postoperative pain score (*p* < 0.001), longer operation time (*p* = 0.001), and higher FKMIP (*p* < 0.001).

**Table 2:** The associations of baseline and peri-operative characteristics with complications

		Complications		*P* value
		No (n = 1136)	Yes (n = 102)	
Median age (range)		50.0 (39.0, 59.0)	48.0 (40.0, 57.0)	0.738^b^
Sex	Female	579 (51.0%)	41 (40.2%)	0.039*^a^
	Male	557 (49.0%)	61 (59.8%)	
Hemorrhoids grade	III	807 (71.0%)	48 (47.1%)	< 0.001*^a^
	IV	329 (29.0%)	54 (52.9%)	
ASA grade	I	398 (35.0%)	33 (32.4%)	0.277^a^
	II	724 (63.7%)	66 (64.7%)	
	III	14 (1.2%)	3 (2.9%)	
Preoperative hemoglobin (g/dl)		13.6 (12.4, 14.8)	14.1 (12.3, 15.4)	0.296
Postoperative pain score		3.0 (2.0, 5.0)	4.0 (2.0, 6.0)	< 0.001*^b^
Operation time (min)		18.0 (13.0, 25.0)	20.0 (15.0, 29.0)	0.001*^b^
FKMIP	0	727 (64.0%)	46 (45.1%)	< 0.001*^b^
	1	310 (27.3%)	31 (30.4%)	
	2	71 (6.3%)	17 (16.7%)	
	3	22 (1.9%)	6 (5.9%)	
	4	5 (0.4%)	1 (1.0%)	
	5	1 (0.1%)	1 (1.0%)	

Categorical variables were presented by count and percentage. Continuous variables were presented by median and *interquartile range* (IQR). ^a^Fisher’s exact test. ^b^ Mann–Whitney U test. *Pis significant. Postoperative pain score: the pain score on the morning of the 1^st^ postoperative day, ketoprofen (30 mg IM/per time), morphine (5 mg IV/per time)

American Society of Anesthesiologists (ASA); Frequency of ketoprofen or morphine injection postoperatively (FKMIP); intramuscular (IM); intravenous (IV)

The above associations with complications were further verified in logistic regression analyses in Table 3 (where frequency of ketoprofen or morphine injection postoperatively was not included due to its collinearity with post-operative pain score).

**Table 3:** The influence factors for all complications, urine retention, and delayed bleeding

	All complications		Urine retention		Delayed bleeding	
	Odds ratio (95% CI)	*P* value	Odds ratio (95% CI)	*P* value	Odds ratio (95% CI)	*P* value
**Univariable analysis**						
Age of 65 years or more vs. age < 65 years	1.15 (0.65, 2.04)	0.631	3.17 (1.27, 7.90)	0.013*	0.59 (0.25, 1.39)	0.231
Male vs. female	1.55 (1.02, 2.34)	0.038*	2.18 (0.88, 5.38)	0.091	1.40 (0.86, 2.27)	0.176
Hemorrhoids grade IV vs. III	2.76 (1.83, 4.16)	< 0.001*	14.82 (4.36, 50.40)	< 0.001*	1.91 (1.18, 3.10)	0.009*
ASA grade I	reference group		reference group		reference group	
ASA grade II	1.10 (0.71, 1.70)	0.670	2.35 (0.79, 7.02)	0.127	0.96 (0.58, 1.59)	0.867
ASA grade III	When	0.151	6.67 (0.71, 63.14)	0.098	2.17 (0.47, 10.00)	0.322
Preoperative hemoglobin (g/dl)	0.98 (0.89, 1.07)	0.614	0.75 (0.65, 0.87)	< 0.001*	1.06 (0.94, 1.19)	0.368
Operation time (min)	1.04 (1.02, 1.06)	0.001*	1.08 (1.04, 1.12)	< 0.001*	1.02 (0.99, 1.04)	0.215
Postoperative pain score	1.26 (1.14, 1.40)	< 0.001*	1.54 (1.25, 1.88)	< 0.001*	1.13 (1.00, 1.28)	0.046*
**Multivariable analysis**						
Age of 65 years or more vs. age < 65 years			3.54 (1.33, 9.42)	0.011*		
Male vs. female	1.51 (0.99, 2.29)	0.055	3.08 (1.18, 8.05)	0.022*		
Hemorrhoids grade IV vs. III	2.26 (1.47, 3.49)	< 0.001*	8.31 (2.32, 29.73)	0.001*	1.72 (1.03, 2.88)	0.037*
Preoperative hemoglobin (g/dl)			0.76 (0.66, 0.89)	< 0.001*		
Postoperative pain score	1.18 (1.06, 1.31)	0.003*	1.40 (1.09, 1.79)	0.008*	1.09 (0.95, 1.24)	0.224

The analyses are performed using univariable and multivariable logistic regression models. *P < 0.05 indicates the association with the specified complication obtained statistically significant

confidence interval (CI); American Society of Anesthesiologists (ASA)

In the univariable logistic regression, male (vs female, OR: 1.55, 95% CI: 1.02–2.34), Hemorrhoids grade IV (vs Hemorrhoids grade III, OR: 2.76, 95% CI: 1.83–4.16), operation time (OR: 1.04, 95% CI: 1.02–1.06), postoperative pain score (OR: 1.26, 95% CI: 1.14–1.40) had significantly higher risk for all complications.

The multivariable logistic regression showed that hemorrhoids grade IV (vs Hemorrhoids grade III, aOR: 2.26, 95% CI: 1.47–3.49) and higher postoperative pain score (aOR: 1.18, 95% CI: 1.06–1.31) has significantly higher risk in all complication.

### Factors associated with postoperative urine retention and delayed bleeding

Patients who were 65 years old or older (vs age < 65 years, OR: 1.15, 95% CI: 0.65–2.04), Hemorrhoids grade IV (vs Hemorrhoids grade III, OR:14.82, 95% CI: 4.36–05.40), lower preoperative hemoglobin (OR:0.75, 95% CI: 0.65–0.87), longer operation time (OR: 1.08, 95% CI: 1.04–1.12), higher postoperative pain score (OR: 1.54, 95% CI: 1.25–1.88) have significantly higher risk in urine retention. Hemorrhoids grade IV (vs Hemorrhoids grade III, OR: 1.91, 95% CI: 1.18–3.10), higher postoperative pain score (OR 1.13, 95% CI 1.00–1.28) has significantly higher risk in delayed bleeding.

For urine retention, the factors with *p* value < 0.1 in univariable analyses were included in the multivariable analyses. The variable operation time was excluded, for its influence did not remain statistically significant in multivariable analyses.

The final multivariable model showed that patients with age ≥ 65 years (vs younger patients, aOR = 3.54, 95% CI: 1.33–9.42), males (vs females, aOR = 3.08, 95% CI:1.18–8.05), and those with hemorrhoids grade IV (vs hemorrhoids grade III, aOR = 8.31, 95% CI: 2.32–29.73), were more likely to occur postoperative urine retention. (Table 3).

The associations of hemorrhoids grade and postoperative pain score with delayed bleeding obtained statistically significant in univariable analyses. The association of hemorrhoids grade remained statistically significant in multivariable logistic regression model. The patients with hemorrhoids grade of IV were more likely to occur delayed bleeding than those with hemorrhoids grade of III (adjusted OR of 1.72, 95% CI: 1.03–2.88, *p* value = 0.037), with the adjustment of postoperative pain score (*p* = 0.224) (Table 3).

## Discussion

Surgeons and elderly patients were most concerned about complications or surgical risk in hemorrhoidectomy. In this study, all patients were followed for at least six weeks after surgery. Although the ASA grade was significantly higher in the elderly than in the younger group, there was no severe morbidity or mortality during or after surgery. It might be due to a relatively minor operation for patients undergoing hemorrhoidectomy and short duration of operative and anesthetic times. Besides, concerning the postoperative outcomes in both groups, the total complications rate, re-admission rate, and re-operation rate in both groups had no significantly statistical difference.

The majority of patients who are re-admitted or re-operative in both groups suffered from the complication of delayed postoperative hemorrhage. No immediate bleeding (within 24 h postoperatively) was noted in both groups. As we know, delayed hemorrhage after hemorrhoidectomy is described as a complication that cannot be completely prevented [11]. The reason of delayed bleeding may be local sepsis of the ligated pedicle, erosion of the suture or oozing from edges of an unhealed dehiscent wound [11–13]. Several series reported the incidence of delayed bleeding after conventional or LigaSure hemorrhoidectomy was between 0.9 and 10% [12–18]. Our incidence of delayed bleeding in this study was 5.73% (71/1238). The majority of patients with delayed bleeding can be managed successfully with conservative treatment [12–15]. Guilherme de Almeida Santos et al. had reported 2,840 patients submitted to hemorrhoidectomy, 23 (0.8%) developed severe anal bleeding, which required surgical intervention [19]. In our series, three patients (3/1238, 0.24%) developed delayed bleeding requiring reoperation. We had described in our previous publication that although the original design of LigaSure device for hemorrhoidectomy was performed in a sutureless fashion, some patients may present delayed bleeding from the edges of dehiscent wound. [8, 12] Thus, in the recent two years, we routinely put some stitches on the LigaSure welting line. Although we had not yet collected the data, we really felt improvement in delayed bleeding occurrence.

The contributions of six characteristics (age, sex, ASA grade, preoperative hemoglobin, operation time, and postoperative pain score) to postoperative complications were evaluated in this study. The results in multivariable analysis revealed that age was not a risk factor but postoperative pain score was the factor significantly associated with a risk of postoperative complications. Regarding the risk of postoperative urine retention in this study, aged 65 years or more, male (compared to female), or postoperative pain score was associated with a higher risk of postoperative urine retention. It revealed that urine retention was a more prevalent and higher risk in the elderly and male patients than the younger ones, which might also explain why male have higher complications after hemorrhoidectomy than female patients in this study (*p* = 0.039, Table 2). Patients with a higher index of pain after hemorrhoidectomy is more likely to have urinary retention. The incidence of urine retention after the LigaSure hemorrhoidectomy for chronic hemorrhoids had been reported to range from 0 to 11.1 percent in different series. [8–14, 20–25] In this study, both groups received the same anesthetic method and urinary catheterization was not performed during the operation. Besides, the postoperative pain scores in both groups were not different (*p* = 0.139, Table 1). Fortunately, urinary retention after hemorrhoidectomy is a relatively minor and transient complication. Patients could urinate spontaneously after the removal of the urinary catheter.

Elbetti et al. had reported an increase in the number of pathological piles treated corresponding to an increase in the need of analgesics regardless of the procedure performed [26]. In our study, we performed Ferguson closed hemorrhoidectomy in patients. It was necessary to perform excisions of three hemorrhoidal cushions. Thus, all patients had three wounds located at left lateral, right anterior and right posterior anal positions.

In exploring whether delayed bleeding occurred, the postoperative pain score was significant in univariable analysis and associated with this complication while showing no significance in multivariable analysis. The incidence of postoperative bleeding after the LigaSure hemorrhoidectomy have been reported to range from 0 to 10% in different series [8–14, 20–24]. In this study, all patients taking anticoagulants were instructed to stop taking for one week before and after surgery. The results showed that the risk of postoperative bleeding was not increased in elderly patients. Conversely, the elderly patients tended to have less delayed bleeding though there was no statistical significance. The reason might be that most elderly patients have retired and can get more rest after surgery.

The limitations are as follows: This is a retrospective study for short-term outcomes.

## Conclusion

This study indicated that hemorrhoidectomy performed in elderly population is safe and effective. In addition, no significant difference in short-term surgical outcomes and total complications was founded between the elder and younger patients. A prospective trial with longer follow-up is needed to confirm these results.

## Data Availability

The datasets used during the current study are available from the corresponding author on reasonable request.

## Abbreviations

FKMIP: Frequency of ketoprofen or morphine injection postoperatively
IQR: Inter-quartile range
